# Hypoxia-inducible factor 2-alpha-dependent induction of IL-6 protects the heart from ischemia/reperfusion injury

**DOI:** 10.18632/aging.202276

**Published:** 2021-01-10

**Authors:** Jia-Wei Wu, Hao Hu, Dan Li, Li-Kun Ma

**Affiliations:** 1 Department of Cardiology, The First Affiliated Hospital of USTC, Division of Life Science and Medicine, University of Science and Technology of China, Hefei 230001, China

**Keywords:** myocardial ischemia-reperfusion injury, HIF2α, IL-6

## Abstract

Myocardial ischemia-reperfusion injury (MIRI) results in increased myocardial infarct size and leads to poor clinical outcomes. Hypoxia-inducible factor 2-alpha (HIF2α) exerts myocardial protective effects during MIRI through as yet unclear mechanisms. Here, we show that knockdown of HIF2α with cardiotropic recombinant adeno-associated virus serotype 9 (rAAV9) in mouse hearts significantly increased the infarct sizes during myocardial ischemia/reperfusion (MI/R). In addition, HIF2α transcriptionally regulated the expression of interleukin 6 (IL-6) in cardiomyocytes to elicit cardioprotection. Likewise, IL-6 deficiency aggravated MIRI, while treatment with recombinant IL-6 had cardioprotective effects and rescued the mice with HIF2α knockdown. Furthermore, IL-6 treatment significantly activated the PI3K/Akt and STAT3 signaling pathways in the myocardium during MI/R, and the specific inhibitors wortmannin (specific phosphoinositide 3-kinase inhibitor) and Stattic (specific STAT3 inhibitor) substantially abolished HIF2α/IL-6-induced cardioprotection. These studies suggest that HIF2α transcription regulates the expression of IL-6 in cardiomyocytes and plays a protective role during MI/R.

## INTRODUCTION

Acute myocardial infarction is a critical cardiovascular disease with a high mortality rate [1]. Effective and prompt reperfusion of the ischemic myocardium is key for saving the heart. Reperfusion effectively reduces the myocardial infarction area, improves long-term heart function, and reduces mortality [2]. However, this process can also be accompanied by ischemia-reperfusion injury, which further increases the myocardial infarction area and leads to a poor clinical prognosis [3–5]. Therefore, an endogenous approach against ischemia-reperfusion injury may effectively help prevent and treat ischemic cardiomyopathy [6].

Hypoxia-inducible factors (HIFs) are nuclear transcription complexes that mediate the hypoxia response in mammalian cells and are the most important gene transcription regulators under hypoxic conditions [7]. The HIF2α functional subunit regulates HIF- 2 activity [8]. HIF2α plays a protective role during myocardial ischemia-reperfusion [9, 10]. HIF2α has also been shown to diminish MIRI by inducing AREG expression in cardiomyocytes. The myocardial necrotic area in HIF2α knockout mice was larger than that in wild-type controls [9]. However, the mechanism underlying the effects of HIF2α during myocardial ischemia-reperfusion has not been fully elucidated.

IL-6, a pleiotropic cytokine, regulates acute immune responses during inflammation and hematopoiesis. Moreover, IL-6 acts against infection during the immune response [11]. The final effects of the IL-6 signaling pathway depend on the duration of the signal and the downstream activation signal. IL-6 often exerts an anti-inflammatory effect in the acute phase of inflammation. However, with the progression of the inflammatory response, the effect becomes gradually proinflammatory and can have adverse effects on the body. In the acute phase, IL-6 preserves cardiac tissue by inducing an antiapoptotic program in the myocytes and triggering a preconditioning response [12, 13]. When IL-6 signaling continues chronically, these protective responses become pathogenic and inhibit myocardial function. As a result, decreased contractility and LV enlargement occur [14–17]. Clinical studies have shown a correlation between the area of myocardial necrosis and the plasma IL-6 levels in patients with acute myocardial infarction [18], suggesting an association between IL-6 levels and MIRI. Elevated IL-6 serum levels in patients with acute coronary heart disease may be predictive of poor outcomes [19–22]. Moreover, myocardial secretion of IL-6 plays an important role in cardiac dysfunction due to ischemia-reperfusion injury [23]. However, the upstream regulatory mechanism of IL-6 involved in MIRI has not been elucidated.

An association between HIF2α and IL-6 has also been discovered in cancers and osteoarthritis [24–27]. IL-6 is the direct target gene of HIF2α in mouse articular chondrocytes and can induce the upregulation of IL-6-specific receptors. However, no studies have focused on the association between HIF2α and IL-6 during ischemia-reperfusion injury in acute myocardial infarction.

We aimed to answer the following questions: (i) Does HIF2α exert a protective effect during myocardial ischemia-reperfusion? (ii) Is IL-6 involved in the protective role of HIF2α in myocardial ischemia-reperfusion?

## RESULTS

### HIF2α has cardioprotective effects in cardiomyocytes during simulated hypoxia/reperfusion

**Figure 1:**
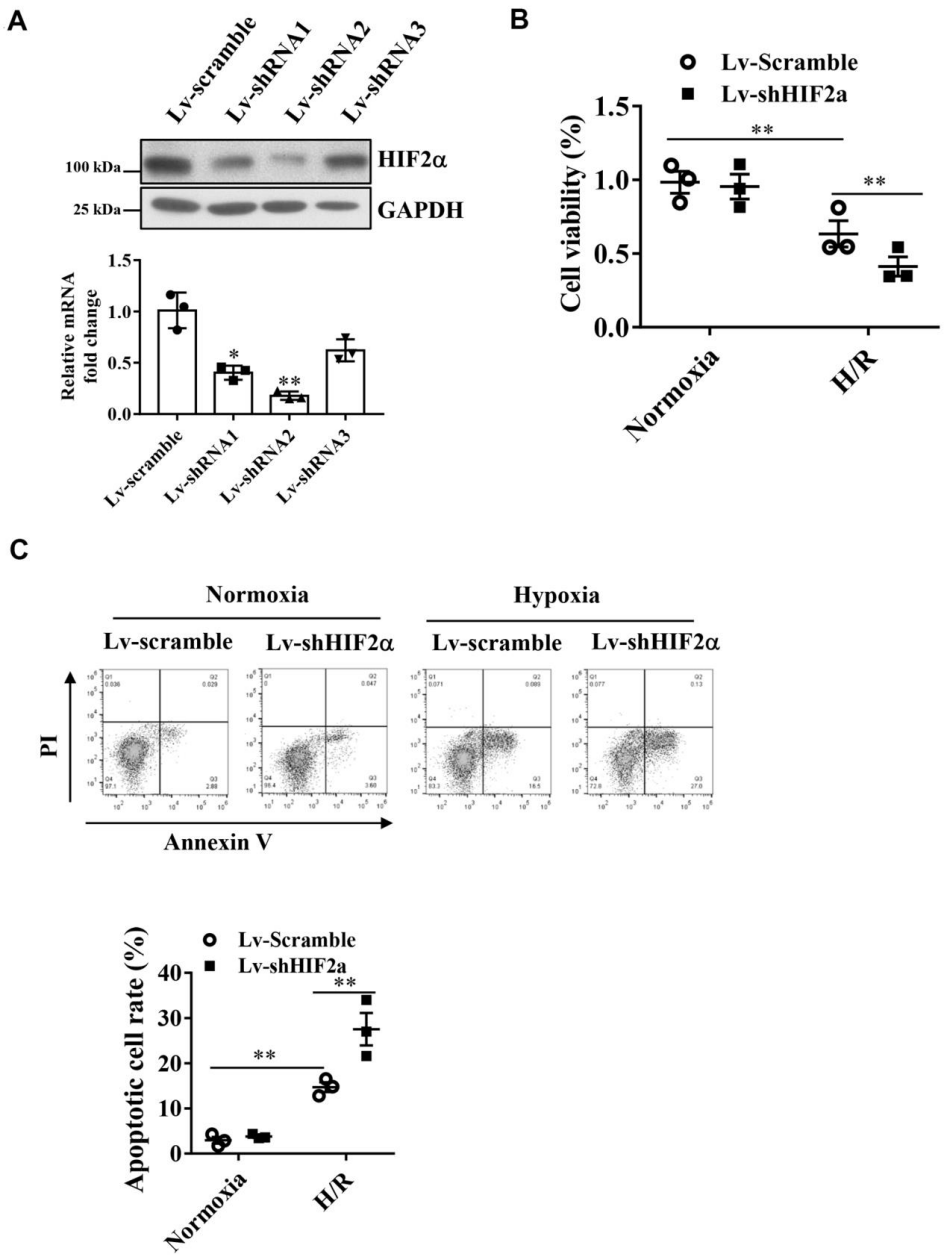
**Effects of HIF2α on cell apoptosis and cell survival of cardiomyocytes during hypoxia.** (**A**) qPCR and Western blot analysis of the efficiency of HIF2α gene- specific downregulation of lentiviral vectors. (**B**) CCK-8 detection of the effect of downregulation of the HIF2α gene on early cell apoptosis and cell survival. (**C**) Application of FACS-Annexin V/PI double staining to detect the effect of downregulation of the HIF2α gene on cell apoptosis. n = 3 per group. Data represent the mean ± SEM. **P<0.01 versus the indicated group.

Studies have shown that HIF2α plays a protective role during MIRI, and therefore, we investigated the underlying mechanisms. To assess the effect of HIF2α in H9c2 cardiomyocytes during hypoxia/reoxygenation (H/R), we generated a lentiviral vector to knock down the endogenous HIF2α gene in H9c2 cardiomyocytes. qPCR and Western blot results showed that the downregulation efficiency of shRNA 1 and 2 reached more than 60% (Figure 1A).

After knocking down the endogenous HIF2α gene, we subjected H9c2 cardiomyocytes to H/R treatment. Our results showed that the apoptotic rate of cardiomyocytes increased significantly after H/R. Moreover, after knockdown of the HIF2α gene in H9c2 cells, the proportion of apoptotic cells increased further (Figure 1C). In addition, we found that knocking down the endogenous HIF2α gene reduced cell survival after H/R (Figure 1B), suggesting that HIF2α confers cardioprotective effects on H9c2 cardiomyocytes during I/R.

### HIF2α regulates the IL-6 level in cardiomyocytes during simulated hypoxia/reperfusion

**Figure 2:**
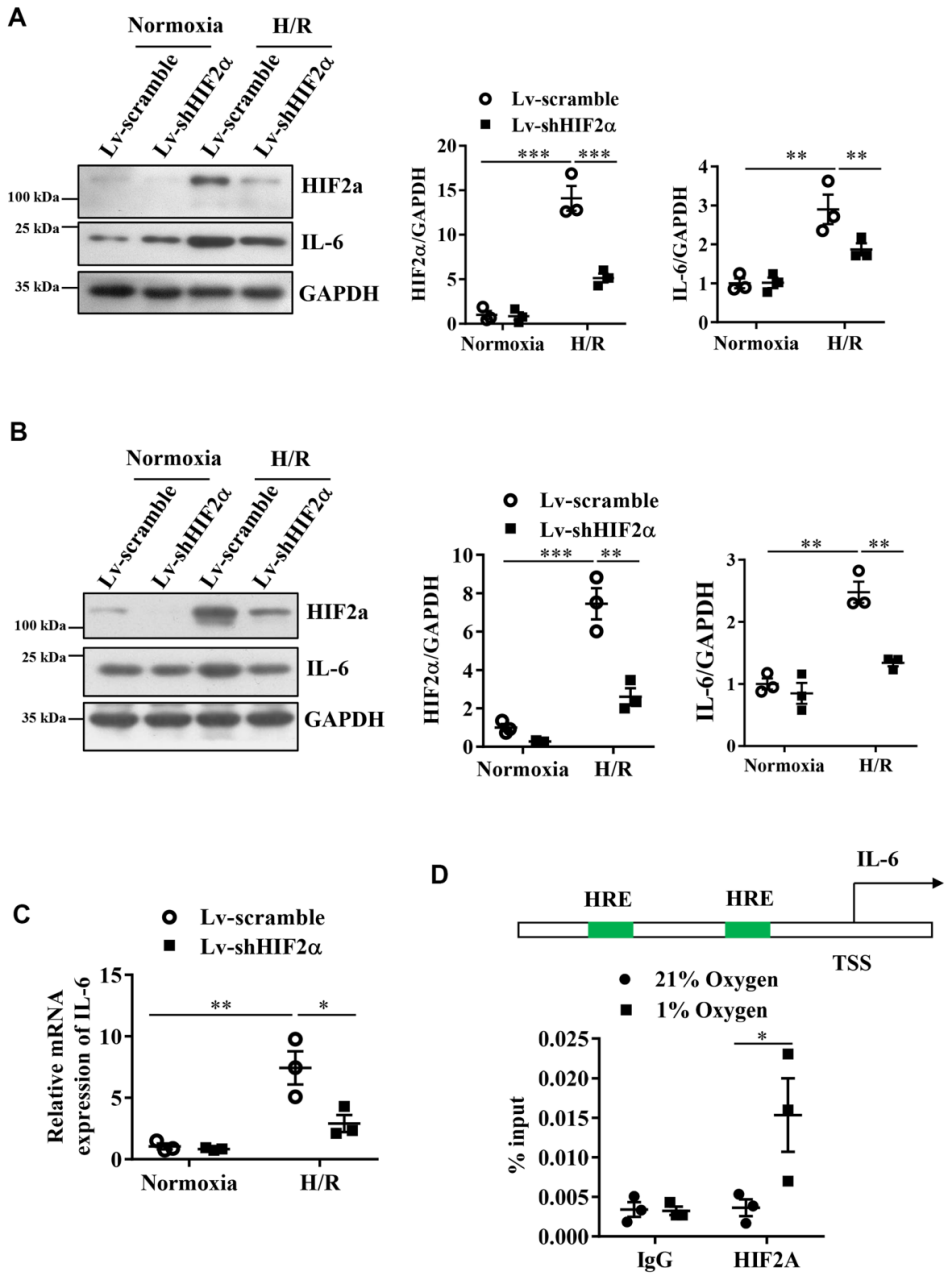
**Expression of IL-6 during the H/R process in cardiomyocytes after knocking down endogenous HIF2α.** (**A**) H9c2 myocardial cell line: Western blot to detect the expression level of IL-6 after knocking down endogenous HIF2α. (**B**) AC16 human cardiomyocyte line: Western blot to detect the expression level of IL-6 after knocking down endogenous HIF2α. (**C**) H9c2 cardiomyocyte cell line: qPCR to detect the expression level of IL-6 mRNA after knocking down endogenous HIF2α. (**D**) The results of ChIP: Diagram of the IL-6 promoter. Quantitative PCR analysis of anti- HIF2α antibody-precipitated and control IgG antibody-precipitated chromatin from cardiomyocytes cultured in 21 or 1% O_2_. HRE: hypoxia response element. n = 3 per group. Data represent the mean ± SEM. *P<0.05, **P<0.01, ***P<0.001 versus the indicated group.

Our results showed that compared with the cells in a normoxic environment, those under H/R conditions expressed significantly increased levels of HIF2α and IL-6 proteins. This result suggests that H/R stimulates the expression of HIF2α and IL-6. After knockdown of endogenous HIF2α, the IL-6 protein level was also low in the mice (Figure 2A).

Based on the finding that HIF2α regulates IL-6 protein levels in the murine *in vitro* model of H/R, we performed studies in AC16 human cardiomyocytes to verify whether the observed changes in murine IL-6 levels also occur in AC16 human cardiomyocytes. Our results also showed that the H/R process stimulated the expression of IL-6 in AC16 human cardiomyocytes. After knocking down endogenous HIF2α, we observed a reduction in the protein expression levels of HIF2α and IL-6 in these AC16 human cardiomyocytes during H/R (Figure 2B). Additionally, we used qPCR to detect the expression of IL-6 mRNA during H/R. Our results were consistent with those of the Western blots. The IL-6 mRNA level in H9c2 cardiomyocytes increased during H/R. However, after knockdown of the HIF2α gene, the IL-6 mRNA levels decreased significantly (Figure 2C). Together, these data suggest that HIF2α regulates IL-6 levels in cardiomyocytes during simulated H/R conditions.

### HIF2α transcriptionally regulates IL-6 expression in cardiomyocytes

Next, a chromatin immunoprecipitation experiment was performed to further verify that HIF2α has a direct regulatory effect on IL-6 at the transcriptional level. First, by sequence analysis, we found two original HIF transcription factor response elements (HRE) in the IL-6 gene promoter regions (Figure 2D). The results from our chromatin immunoprecipitation experiments showed that the DNA from the promoter region of the IL-6 gene recruited by the HIF2α antibody showed a greater enhancement under hypoxic conditions than under normoxic conditions. However, we found no significant differences in terms of the DNA of the promoter region pulled down by the IgG antibody (Figure 2D). Taken together, our results indicate that HIF2α directly regulates the transcription of IL-6 in cardiomyocytes.

### HIF2α confers cardioprotective effects under myocardial I/R injury

**Figure 3:**
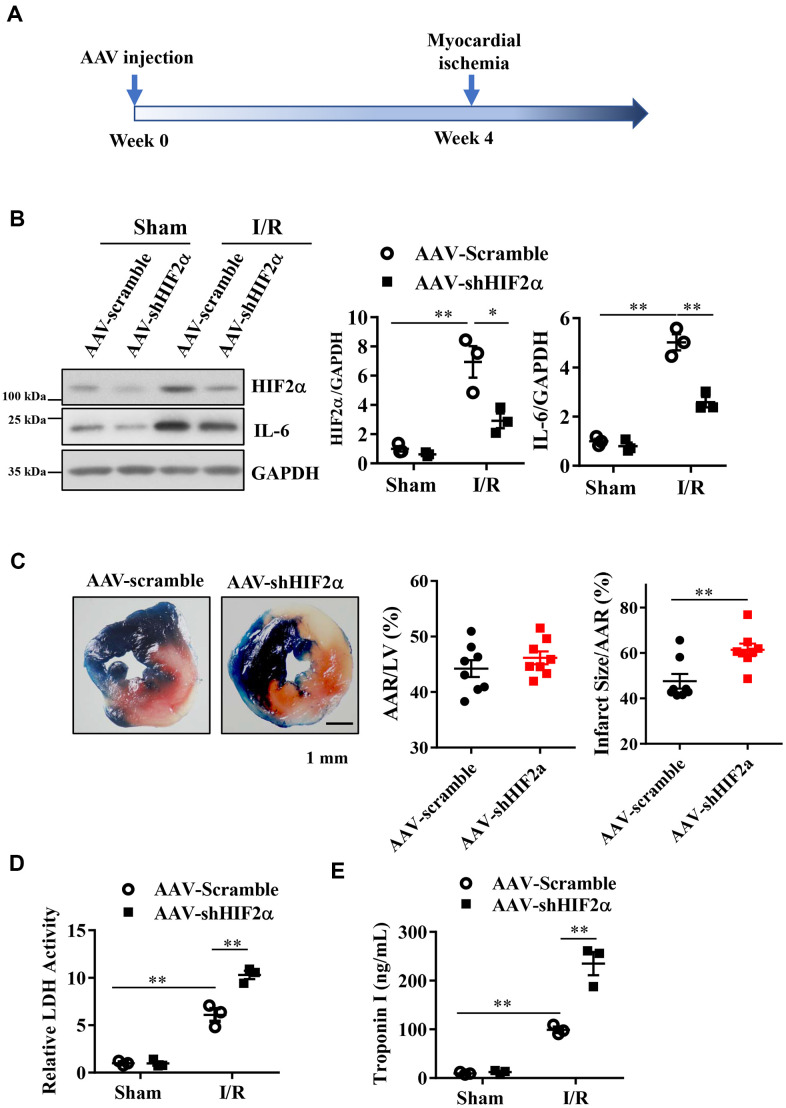
**The effect of *in vivo* HIF2α knockdown on myocardial infarction during acute myocardial ischemia/reperfusion.** (**A**) Myocardial I/R model was established 4 weeks after AAV injection. (**B**) Western blots were performed to examine the HIF2α and IL-6 protein expression after HIF2α knockdown *in vivo*. n = 3 per group. (**C**) TTC and Evans blue staining after mouse heart I/R. n = 8 per group. (**D**) Effect of HIF2α knockdown on myocardial LDH release subsequent to I/R. n = 5 per group. (**E**) Effect of HIF2α knockdown on myocardial TnI release subsequent to I/R. n = 5 per group. Data represent the mean ± SEM. **P<0.01 versus the indicated group.

Then, we conducted *in vivo* studies in mice to investigate whether HIF2α confers protective effects on MIRI. We constructed a type 9 adeno-associated virus vector (AAV9- shHIF2α) that specifically downregulates HIF2α. Four weeks after injection of the AAV (5*10^10 PFU) vector via the mouse tail vein, we ligated the LAD coronary artery in each mouse to establish an *in vivo* myocardial ischemia/reperfusion (I/R) model (Figure 3A). Our results showed that the expression levels of HIF2α and IL-6 in the myocardium increased significantly after myocardial I/R, and this change was reversed with AAV9-shHIF2α delivery (Figure 3B). Additionally, we isolated the hearts of mice 24 h after reperfusion and extracted the left ventricular blood for LDH and TnI determination, while we used TTC and Evans blue staining to evaluate the myocardial infarction areas. Our results showed no significant differences in terms of the myocardial risk areas between the sham groups. However, the myocardial infarction area in the shHIF2α knockdown group was significantly increased compared to that in the control group (Figure 3C). We also found that the myocardial injury markers LDH and TnI were significantly increased in the I/R group and were further enhanced with HIF2α knockdown (Figure 3D, 3E). These results suggest that HIF2α deficiency *in vivo* exacerbates MIRI.

### IL-6 knockdown aggravates myocardial I/R injury

**Figure 4:**
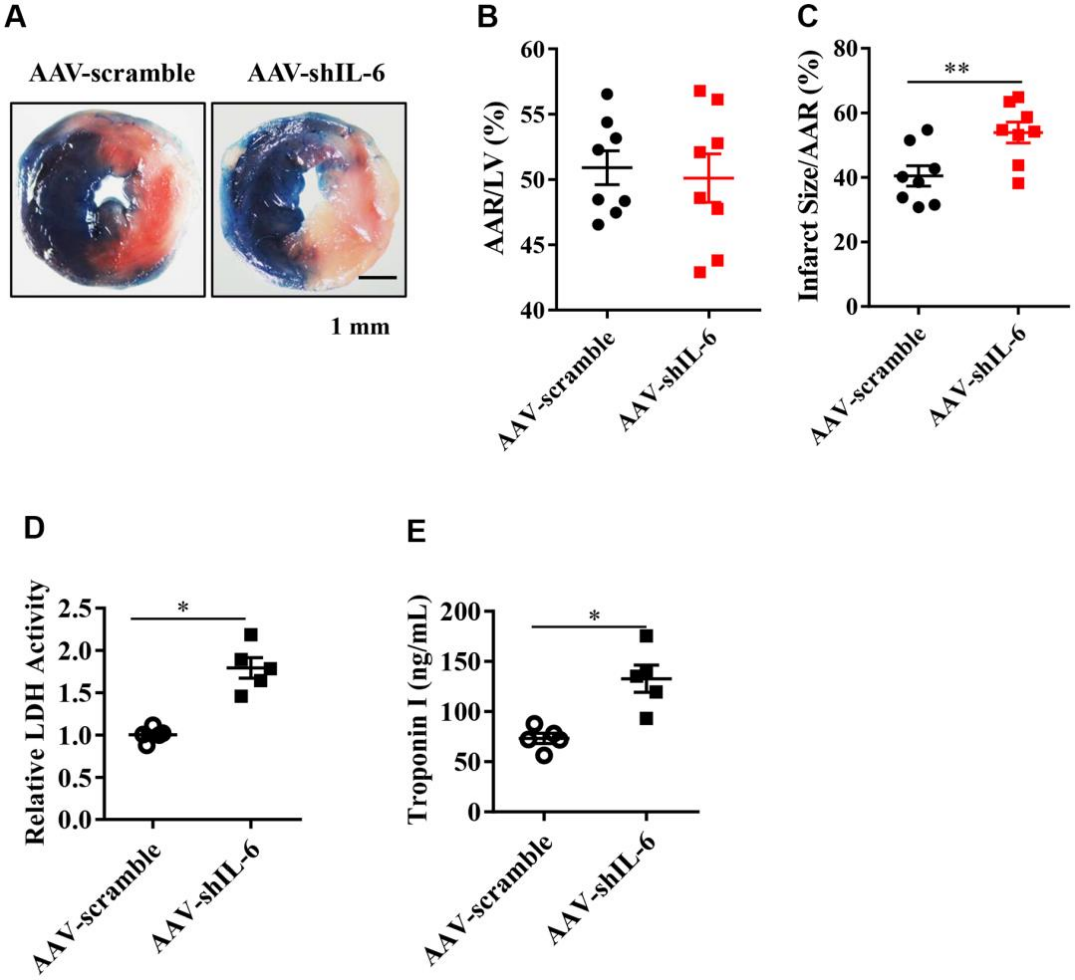
**The effect of *in vivo* IL-6 knockdown on the myocardial infarction area and markers of myocardial injury.** (**A**) TTC and Evans blue staining after mouse heart I/R. (**B**) Mice in the AAV-scramble group and AAV-shIL-6 group experienced similar cardiac risk areas. n = 8 per group. (**C**) The range of myocardial infarction in the AAV- shIL-6 group was larger than that in the AAV-scramble group. n = 8 per group. (**D**) Effect of IL-6 knockdown on the LDH release subsequent to I/R. n = 5 per group. (**E**) Effect of IL-6 knockdown on the troponin I release subsequent to I/R. n = 5 per group. Data represent the mean ± SEM. *P<0.05, **P<0.01 versus the indicated group.

Next, we sought to determine whether IL-6 is involved in the protective effects of HIF2α during myocardial I/R. First, we constructed a type 9 adeno-associated virus vector (AAV9-shIL-6) that specifically downregulates the IL-6 gene. Our results showed that both the mice in the AAV-scramble and AAV-shIL-6 groups had similar cardiac risk areas (Figure 4A, 4B). However, the mice in the AAV-shIL-6 group had larger myocardial infarction ranges than the mice in the AAV-scramble group (Figure 4A, 4C), with higher LDH and TnI values (Figure 4D, 4E). These findings indicate that *in vivo* knockdown of IL-6 increases the range of myocardial infarction in mice. That is, IL-6 knockdown *in vivo* exacerbates MIRI. Our observations confirmed that IL-6 is important for the protective effects of HIF2α during myocardial I/R.

### Recombinant IL-6 treatment diminishes I/R injury in IL-6 knockdown mice

**Figure 5:**
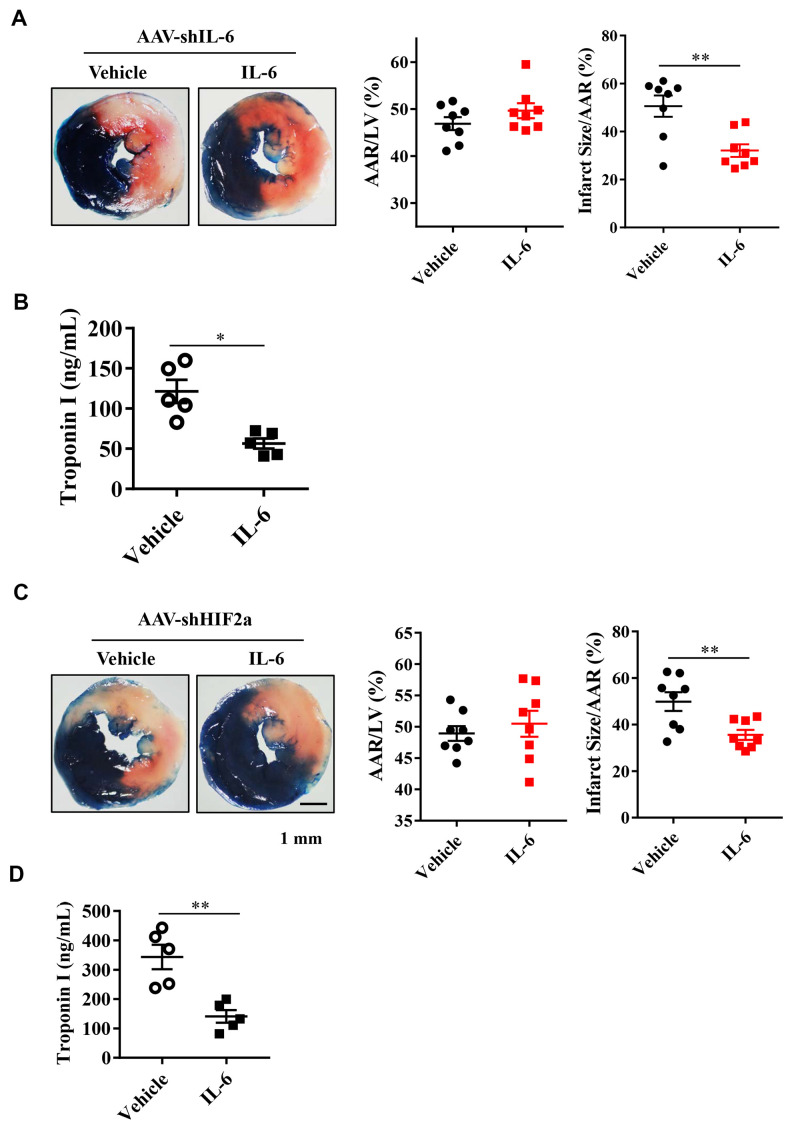
**Effect of exogenous IL-6 supplementation on myocardial infarct size and troponin I in endogenous IL-6 knockdown mice or HIF2α deficiency mice.** (**A**) Representative image and quantitative analysis of TTC and Evans blue staining after mouse heart I/R with IL-6 supplementation in IL-6 deficiency mice. n = 8 per group. (**B**) The results of troponin I release subsequent to I/R. n = 5 per group. (**C**) Representative image and quantitative analysis of TTC and Evans blue staining after mouse heart I/R with IL-6 supplementation in HIF2α deficiency mice. n = 8 per group. (**D**) The results of troponin I analysis. n = 5 per group. Data represent the mean± SEM. *P<0.05, **P<0.01 versus the indicated group.

Based on our studies in mice with the *IL-6* gene downregulated in cardiomyocytes, we hypothesized that treatment with recombinant IL-6 could provide cardioprotection from I/R injury. Mice were exposed to I/R injury with intravenous injection of IL-6 at a dose of 10 μg/kg or vehicle 15 min before the onset of myocardial ischemia. After a 30-min exposure to myocardial ischemia followed by 24 h of reperfusion, we found significantly reduced myocardial infarct sizes and troponin I serum levels in the mice treated with recombinant IL-6 compared with those treated with vehicle (Figure 5A, 5B). These findings suggest that supplementation with recombinant IL-6 reduced the extent of myocardial infarction.

### IL-6 treatment rescues the HIF2α-knockdown mice

Next, we investigated whether treatment with recombinant IL-6 rescues mice with downregulated HIF2α. After knocking down the HIF2α gene, we treated mice with 10 μg/kg recombinant IL-6. The results of Evans blue staining showed similar cardiac risk areas after subsequent exposure to 30 min of ischemia and 24 h of reperfusion between the IL-6-treated and control mice (Figure 5C). However, the area of myocardial infarction and the level of troponin I were both significantly lower in the IL-6-treated mice than in the control mice (Figure 5C, 5D). Thus, recombinant IL-6 treatment is associated with attenuated MIRIs in the HIF2α-knockdown mice.

### The PI3K/Akt and STAT3 signaling pathways are involved in HIF2α/IL-6- induced cardioprotection

**Figure 6:**
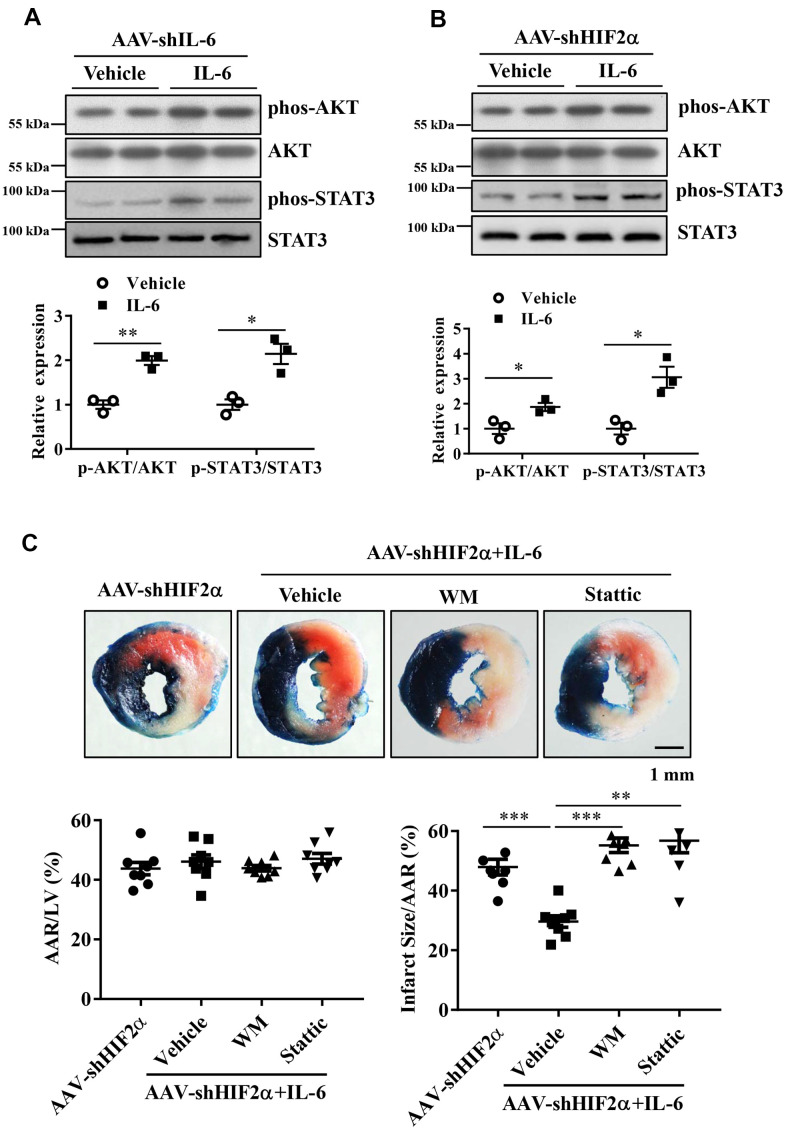
**The roles of the PI3K/Akt and STAT3 pathways in the protective effects of HIF2α/IL-6.** (**A**, **B**) Western blotting was performed to determine the protein expression of AKT, Phos-AKT, STAT3 and Phos-STAT3 in myocardium. n = 3 per group. (**C**) Representative image and quantitative analysis of infarct staining with different treatments. n = 8 per group. Data represent the mean ± SEM. *P<0.05, **P<0.01, ***P<0.001 versus the indicated group.

After showing that treatment with recombinant IL-6 reduced the extent of myocardial injury following I/R, we investigated whether administration of recombinant IL-6 activates cardioprotective pathways, e.g., via activation of survival kinases such as Akt and the STAT3 pathway. As described above, we treated the IL-6 knockdown mice and the *HIF2α* deficient mice with recombinant IL-6 or vehicle and examined the phosphorylation of Akt and STAT3. Our results showed that phosphorylated STAT3 (Tyr705) and AKT (Ser473) in the IL-6-treated hearts were significantly increased compared with the control hearts (Figure 6A, 6B).

To determine the roles of PI3K/Akt activation and STAT3 activation in the cardioprotection of HIF2α /IL-6 against myocardial I/R, we administered the PI3K inhibitor wortmannin (1 mg/kg) and the STAT3 inhibitor Stattic (2 mg/kg) to the mice 60 min prior to ischemic insult. Our data indicate that blockade of PI3K/Akt as well as the STAT3 pathway abolished the HIF2α/IL-6-induced cardioprotective effects (Figure 6C). These findings suggest that HIF2α/IL-6 is dependent on PI3K/Akt activation and STAT3 activation to mediate its effects.

## DISCUSSION

In the present study, we address the functional roles of HIF2α in mediating cardioprotective responses through the induction of IL-6. In addition, our study showed that treatment with recombinant IL-6 reduced myocardial injury following MI/R and rescued the HIF2α-knockdown mice. Notably, our further investigation indicates that HIF2α/IL-6 confer cardioprotection through activation of the cardioprotective pathway, e.g., survival kinases such as Akt and STAT3.

Some studies have focused on HIF1α and MIRI, but few have focused on HIF2α and MIRI, and the underlying mechanisms had not been elucidated until now. Others have shown that the area of myocardial necrosis in HIF2α knockdown mice is larger than that in control mice, indicating that HIF2α plays a role in reducing I/R injury [9]. We successively constructed cardiomyocyte H/R models and myocardial I/R models to study the cardioprotective effects of HIF2α. Our *in vitro* experiments showed that hypoxia and reoxygenation induced high expression of HIF2α in cardiomyocytes. Knocking down the endogenous HIF2α gene reduced cell survival after H/R. In addition, *in vivo* experiments indicated that knocking down the HIF2α gene in mice resulted in the worsening of myocardial infarction. These *in vivo* and *in vitro* experiments fully demonstrate a protective role for HIF2α during cardiac I/R.

Interleukin 6 is a pleiotropic cytokine; it plays important roles in many processes, including immune and inflammatory responses, and it regulates the immune response and hematopoiesis. Elevated IL-6 serum levels in patients with coronary heart disease (especially in those with acute myocardial infarction) may be predictive of poor outcomes [19–22]. *In vitro* studies have suggested that IL-6 is involved in MIRI. Moreover, the myocardial secretion of IL-6 plays an important role in cardiac dysfunction due to I/R injury [23]. Kobara et al. treated mice with an IL-6 receptor antagonist (MR16-1) or placebo and ligated coronary arteries to establish an acute myocardial infarction model to observe echocardiographic changes and mouse survival [28]. These researchers found that mice treated with MR16-1 experienced higher left ventricular short-axis shortening rates, lower left ventricular end-diastolic diameters, and significantly higher survival rates than control mice. Other studies have shown that gp130 knockout mice have higher expression of IL-6 binding coreceptors, IL-6, and STAT3 and significantly higher left ventricular dilatations, ruptures, and mortality than control mice [29]. These studies suggest that IL-6 plays an adverse role during acute myocardial infarction in mice. However, other studies have reached different conclusions. Muller et al. used an IL-6 monoclonal antibody to treat mice with acute myocardial infarction and found that the areas of myocardial infarction in the mice treated with the IL-6 monoclonal antibody were larger and the left ventricular ejection fractions were lower than those in the control mice [30]. Moreover, neutrophil infiltration was significantly reduced in the IL-6 monoclonal antibody-treated mice, suggesting that the inflammatory response induced by IL-6 also has cardioprotective effects. In addition, the IL-6/soluble IL-6 receptor (sIL-6R) complex plays a role in reducing apoptosis in cardiomyocytes, and it reduced the percentage of infarcted area to risk area in an I/R model [31]. Thus, the role of IL-6 in myocardial infarction in mice is complex, and cytokines remain a difficult target for treatment. Our study found that IL-6 plays a protective role during myocardial I/R, contrary to the results of previous study [28]. We believe this discrepancy may be related to different research methodologies, especially different animal models (we used a myocardial I/R model, not a myocardial infarction model). The I/R process is thought to trigger a pronounced inflammatory response [32], and IL-6 may produce two completely different effects in acute myocardial infarction and I/R injury models.

The effects of IL-6 during acute myocardial infarction and myocardial I/R have not been fully elucidated. Our study confirmed that IL-6 is involved in the protective effect of HIF2α upon myocardial I/R, a new finding. An association between HIF2α and IL-6 has been reported in cancers and osteoarthritis, and studies have shown that IL-6 is the direct target gene of HIF2α in mouse articular chondrocytes [24]. Additionally, HIF2α can induce the upregulation of IL-6-specific receptors in chondrocytes [25]. However, our series of *in vivo* and *in vitro* experiments confirmed that HIF2α transcription regulates the expression of IL-6 in cardiomyocytes and that it plays a role in preventing MIRI. Wu et al. found a small molecule agonist that can directly bind to the HIF2α protein through *in vitro* compound screening [33]. By changing the conformation of side chains of amino acid residues at the interface of the HIF2α-ARNT dimer, the small molecule agonist can affect the stability of the dimer and regulate its transcriptional activity, providing a new target for the diagnosis and treatment of renal anemia and other ischemic diseases [33]. Further studies with HIF2α protein small molecule agonists should verify whether these molecules affect the cellular expression of IL-6 and reduce MIRI to provide a new target for the prevention and treatment of ischemic cardiomyopathy.

Activation of the PI3K/Akt and STAT3 pathways protects the heart from I/R injury by preventing cardiomyocyte apoptosis. In addition, the activation of the PI3K/Akt and STAT3 pathways can also reduce MIRI by diminishing oxidative stress, inhibiting the inflammatory cascade, and inhibiting apoptosis [34–37]. This finding was confirmed in our study, in which the I/R-increased phosphorylation of Akt (Ser473) and STAT3 (Tyr705) was further enhanced by IL-6. We further confirmed that the activated PI3K/Akt and STAT3 pathways contribute to HIF2α, IL-6-mediated cardioprotection. Further studies are needed to investigate the downstream targets of the PI3K/Akt and STAT3 signaling pathways.

## CONCLUSION

**Figure 7:**
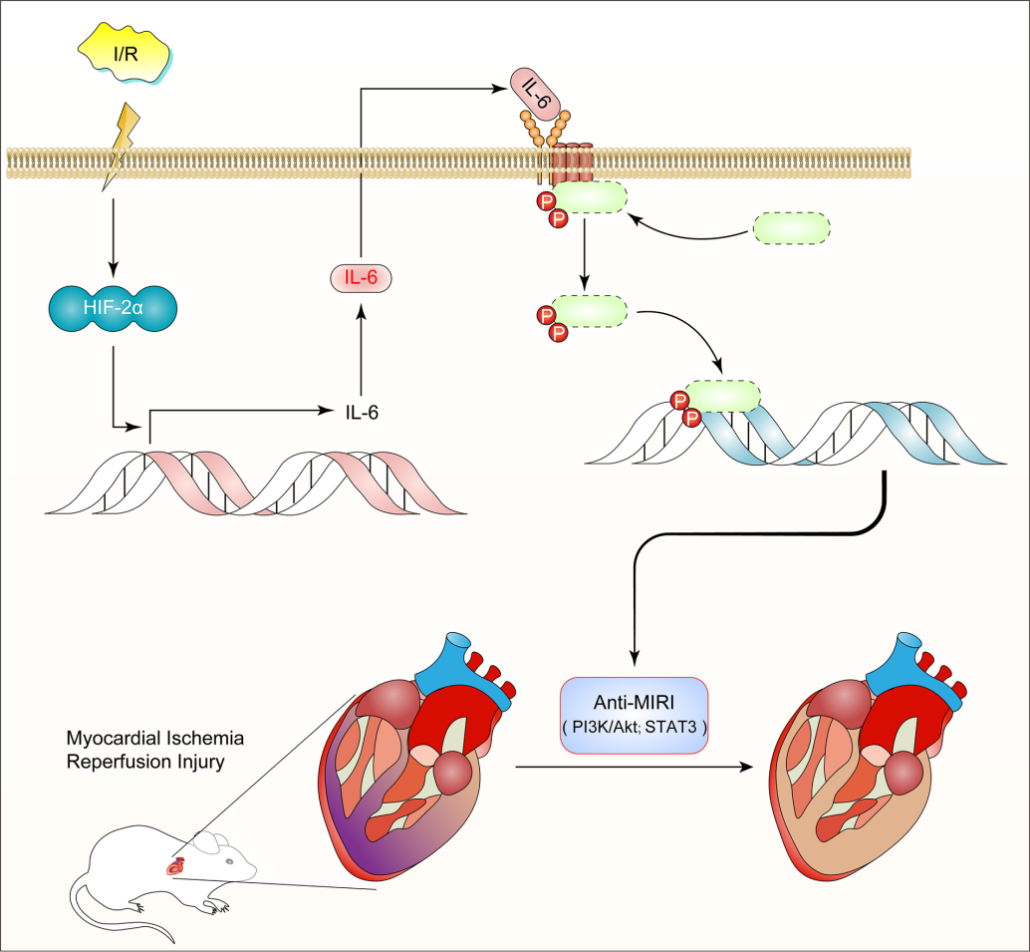
**Diagram of the mechanism: HIF2α -dependent induction of IL-6 protects the heart from ischemia/reperfusion injury.**

Thus, we discovered a previously unidentified role for HIF2α-regulated IL-6 in the myocardium during MIR. Our research suggests that HIF2α transcription regulates the expression of IL-6 in cardiomyocytes and plays a role in preventing MIRI (Figure 7). These findings not only reveal the potential therapeutic value of IL-6 in the protection of the myocardium from ischemic disease but also provide new insight into the molecular mechanisms of the transcriptional factor HIF2α.

## MATERIALS AND METHODS

### Animals

Adult male C57BL/6 mice (8 to 10 weeks old) from the Shanghai Slac Animal Laboratory were maintained and handled in accordance with the Guidelines for Care and Use of Laboratory Animals published by the US National Institutes of Health (NIH Publication, 8^th^ Edition, 2011). The Institutional Review Board of The First Affiliated Hospital of USTC approved all animal procedures (Hefei, China).

### Myocardial I/R model and IL-6 treatment

We performed conventional surgical ligations of the left anterior descending (LAD) coronary arteries in mice as previous studied [38, 39]. Briefly, we anesthetized the mice with intraperitoneal injections of ketamine (50 mg/kg) and pentobarbital sodium (50 mg/kg), orally intubated them, and ventilated them. We maintained their core body temperature constantly at 37° C. Medial sternotomies were then performed using an electrocautery pen to visualize the proximal LAD in each mouse and ligate it. The coronary artery remained occluded for 30 min, after which we cut the sutures and allowed the vessel to reperfuse. We closed the sternum and skin separately and allowed the animals to recover. In the PI3K inhibition and STAT3 inhibition experiments, mice were treated with the PI3K inhibitor wortmannin (1 mg/kg, Selleck Chemicals, No. S2758, USA) and the STAT3 inhibitor Stattic (2 mg/kg, Selleck Chemicals, No. S7024, USA) by intraperitoneal injection 60 min prior to ischemic insult.

### Recombinant IL-6 treatment

Recombinant mouse IL-6 was purchased from R&D Systems (No. 406-ML-025, USA) and dissolved IL-6 in 0.9% NaCl solution. In the IL-6 group, 10 μg/kg recombinant mouse IL-6 was intravenously administered 15 min prior to the onset of myocardial ischemia, whereas the control group received the same volume of 0.9% NaCl over the same period as described previously [34]. There was no difference in mortality between the groups. When the injection was complete, we began the *in situ* myocardial ischemia induction.

### Myocardial area-at-risk and infarct size determination

We measured the area at risk and infarct size. In brief, 24 h after reperfusion, we anesthetized the mice, ventilated them, and catheterized them through the common carotid artery. We performed median sternotomies and religated the LAD coronary arteries in the same location as before. We injected Evans blue dye (1.2 mL of a 4.0% solution; Sigma, No. E2129, USA) into the carotid artery catheter to differentiate the cardiac ischemic zone from the nonischemic zone. We then rapidly excised the hearts and sectioned them serially and incubated the sections in 1.0% 2,3,5-triphenyltetrazolium chloride (Sigma, No. T8877, USA). We weighed each of the five 1-mm thick myocardial slices, and a blinded observer assessed the areas of infarction, risk, and nonischemic left ventricle using computer-assisted planimetry (NIH ImageJ 1.37).

### Cell culture and treatment

The fetal cardiomyocyte-derived H9c2 (American Type Culture Collection) cells were cultured in Dulbecco’s modified Eagle’s medium (Gibco, Grand Island, NY, USA) supplemented with 10% fetal bovine serum, 100 U ml–1 penicillin, 100 μg ml–1 streptomycin and 110 mg ml–1 sodium pyruvate in a humidified atmosphere containing 5% CO_2_ at 37° C. Human cardiomyocytes (HCMs) were purchased from ScienCell (Carlsbad, CA, USA). For the H/R experiments, we first perfused H9c2 cells in normal Hank's solution with a gas mixture of 21% O_2_-5% CO_2_ at 37° C, pH 7.4. For simulation of ischemia, Hank's solution was switched to pH 7.4 at 37° C without glucose or calcium, and then, the cells were aerated with a gas mixture of 95% N_2_-5% CO_2_ for 10 h. To simulate reperfusion, we treated the cells again with normal Hank's solution and a gas mixture of 21% O_2_-5% CO_2_ at 37° C, pH 7.4 for 2.5 h. We included cells under normoxic conditions throughout the experiments as controls.

### Construction of lentivirus expressing shRNA for rat Hif2α

The rat HIF2α RNAi target sequence was 5'-CAGCACTGCTTCAGTGCCAT- GACAA-3'. We used a nonrelated, scrambled RNA sequence without a match in the rat genomic sequence as a control (5'-TCAGTCTTCATGGAACCTT-3'). We generated lentiviruses harboring these RNAi constructs using a lentivirus expression system (Invitrogen Corporation, MA, USA) as published. The constructs were verified by sequencing. We produced viral particles by third-generation packaging in 293FT cells and concentrated lentiviral stocks using ultracentrifugation.

### Viability assays

We assessed cell viability using a cell counting kit-8 (CCK-8, No. C0038, Beyotime Institute of Biotechnology, Haimen, China). We seeded H9c2 cells in 48-well plates for 24 h. Then, we infected the cells with lentivirus for 48 h before subjecting them to H/R treatment. Cells were provided with fresh media, and 10 μl of CCK-8 solution was added to every well. We then incubated the plates under normal conditions for 2 h. We measured optical density values at 470 nm using a microplate reader (Multiskan MK33, Thermolab systems, Helsinki, Finland). We repeated each experiment at least three times.

### Analysis of apoptosis

We used an Annexin V-FITC/PI apoptosis detection kit (Beyotime Institute of Biotechnology, No. C1062S, Haimen, China) to identify the presence of cell apoptosis according to the manufacturer's instructions. After treatments, cells were harvested and resuspended in 200 μl of binding buffer. Then, we incubated the cells with 10 μl of Annexin V-FITC and 5 μl of PI in the dark for 15 min. We assessed the apoptosis rate by flow cytometry analysis.

### Lactate dehydrogenase (LDH) and cardiac troponin-I (TnI) release

We assessed necrotic cell death by measuring supernatant LDH activity. We used a spectrophotometric kit (Nanjing Jiancheng, No. A020-1, China) according to the manufacturer’s instructions. In brief, we collected 20 μl supernatant samples in a 96- well plate and then added 25 μl of matrix buffer and 5 μl of coenzyme I to each well. The mixture was incubated at 37° C for 15 min. Then, we added 25 μl of 2,4- dinitrophenylhydrazine to each well and incubated the cells again at 37° C for 15 min. We finally added 250 μl of 0.4 M NaOH to each well and incubated the plates at room temperature for 5 min. We measured the absorbance value at 450 nm with a spectrophotometer (BioTek, VT, USA) and calculated the LDH activity. We measured plasma TnI levels as an indicator of cardiomyocyte damage using a mouse TnI ELISA kit (Elabscience Biotechnology, No. E-EL-M1203c, China) according to the manufacturer’s instructions.

### Quantitative real-time PCR

We isolated total RNA samples from heart tissues using TRIzol Reagent (Thermo Fisher Scientific, No. 15596026, USA). We performed real-time PCR using SYBR Green detection of PCR products in real time with the ABI Prism 7700 Sequence Detection System (Applied Biosystems, CA, USA) for relative quantification of HIF2α products. The primers used for rat HIF2 α were as follows: forward, 5'- GAGAAGGTGACTCAGCGACA-3'; and reverse, 5'-GTTCTTGCTCCCTCCAACTC-3'. The primers used for rat IL-6 were as follows: forward, 5'-CATTCTGTCTCGAGCCCACC-3'; and reverse, 5'- GCTGGAAGTCTCTTGCGGAG-3'. GAPDH RNA was amplified as a reference standard. The sequences of rat GAPDH primers were as follows: forward, 5'- TGGAGTCTACTGGCGTCTT-3'; reverse, 5'-TGTCATATTTCTCGTGGTTCA-3'. We conducted the reactions in triplicate by heating the reactants to 95° C for 5 min, followed by 40 cycles of 94° C for 30 s, 58° C for 30 s, and 72° C for 30 s.

### Western blot analyses

Left ventricular tissues were homogenized at 4° C with a homogenizer in lysis buffer containing 1% Triton X-100, 0.5% deoxycholate, and 5 mmol/L 2-mercaptoethanol. We scraped cell extracts into lysis buffer containing 20 mmol/L Tris-HCl (pH 7.4), 6 mM urea, and 200 mmol/L potassium chloride with a protease inhibitor cocktail, followed by vigorous vertexing and cooling on ice for 15 min before a 15-min centrifugation at 12,000 g. We then ran the samples on SDS-PAGE. We transferred the proteins on gels onto polyvinylidene fluoride microporous membranes (Bio-Rad, CA, USA) and probed them with primary antibodies against HIF2 α (No. ab109616, Abcam, USA; 1:1000), anti-GAPDH (internal control; No. KC-5G4, Kangcheng, Shanghai, China; 1:8000), anti-IL-6 (No. ab9324, Abcam, USA; 1;1000), anti-AKT (No. #4685, Cell Signaling Technology, USA; 1:2000) and anti-p-AKT (No. #4051, Cell Signaling Technology, USA; 1:2000). The secondary antibodies were coupled with horseradish peroxidase (Cell Signaling Technology, 1:6000). We visualized the immunoreactions using an enhanced ECL detection kit (No. GERPN2209, Amersham Pharmacia Biotech, USA) and then exposed them to film before quantifying them with a video documentation system (Gel Doc 2000, Bio-Rad).

### Chromatin immunoprecipitation

We performed chromatin immunoprecipitation (ChIP) experiments using a Simple ChIPTM Enzymatic Chromatin IP Kit (Cell Signaling Technology) according to the manufacturer’s instructions. Cells were treated with phosphatidylethanolamine and then washed twice with phosphate-buffered saline before crosslinking with 1% formaldehyde at 37° C for 10 min. Next, we isolated nuclear fractions and enzymatically digested the DNA in them. We verified the quality and length of the digested fragments on 1.5% agarose gels. We immunoprecipitated protein-DNA complexes using antibodies against HIF2α (No. NB100-122, Novus Biologicals, USA) and normal rabbit IgG (No. #3900, Cell Signaling Technology, USA). The purified DNA was quantified by qRT-PCR with SYBR Green PCR reagents (Toyobo, Osaka, Japan) to detect the enrichment of the IL-6 promoter with the specific primers 5'- CAGGCATCACTACTGTCTGTG-3' (forward) and 5'-TTCTGACCTA-AGTTTCTTCAG-3' (reverse) and normalized to the total input control.

### AAV9 vector generation and transfection

We prepared AAV9-shHIF2α, AAV9-shIL-6, and AAV9–mCherry vectors as described [40]. We injected 1 x 1012 vg of AAV9-mCherry, AAV9-shHIF2α or AAV9- shIL-6 intravenously into the tail veins of 4- to 5-week-old male C57 mice as described previously [41]. We conducted sham or myocardial ischemia operations 4 weeks after the AAV9 injections.

### Statistical analysis

Data are shown as the mean ± SEM. We used GraphPad Prism® version 6.0 software to perform unpaired, 2-tailed *t* tests for comparisons between 2 groups and ANOVA or repeated ANOVA followed by the Bonferroni post hoc test for multiple comparisons. We considered *P* values < 0.05 as statistically significant.
